# Inhibition of Kpnβ1 mediated nuclear import enhances cisplatin chemosensitivity in cervical cancer

**DOI:** 10.1186/s12885-021-07819-3

**Published:** 2021-02-02

**Authors:** Ru-pin Alicia Chi, Pauline van der Watt, Wei Wei, Michael J. Birrer, Virna D. Leaner

**Affiliations:** 1 Division of Medical Biochemistry & Structural Biology, Department of Integrative Biomedical Sciences, SAMRC/UCT Gynaecological Cancer Research Centre, Faculty of Health Sciences, Institute of Infectious Disease and Molecular Medicine, University of Cape Town, Observatory, Cape Town, 7925 South Africa; 2 Pfizer, Andover, MA 01810 USA; 3 University of Arkansas Medical Sciences, D Winthrop P. Rockefeller Cancer Institute, Little Rock, AR USA

**Keywords:** Cisplatin, INI-43, Nuclear import, p53, NFκB, Cervical cancer

## Abstract

**Background:**

Inhibition of nuclear import via Karyopherin beta 1 (Kpnβ1) shows potential as an anti-cancer approach. This study investigated the use of nuclear import inhibitor, INI-43, in combination with cisplatin.

**Methods:**

Cervical cancer cells were pre-treated with INI-43 before treatment with cisplatin, and MTT cell viability and apoptosis assays performed. Activity and localisation of p53 and NFκB was determined after co-treatment of cells.

**Results:**

Pre-treatment of cervical cancer cells with INI-43 at sublethal concentrations enhanced cisplatin sensitivity, evident through decreased cell viability and enhanced apoptosis. Kpnβ1 knock-down cells similarly displayed increased sensitivity to cisplatin. Combination index determination using the Chou-Talalay method revealed that INI-43 and cisplatin engaged in synergistic interactions. p53 was found to be involved in the cell death response to combination treatment as its inhibition abolished the enhanced cell death observed. INI-43 pre-treatment resulted in moderately stabilized p53 and induced p53 reporter activity, which translated to increased p21 and decreased Mcl-1 upon cisplatin combination treatment. Furthermore, cisplatin treatment led to nuclear import of NFκB, which was diminished upon pre-treatment with INI-43. NFκB reporter activity and expression of NFκB transcriptional targets, cyclin D1, c-Myc and XIAP, showed decreased levels after combination treatment compared to single cisplatin treatment and this associated with enhanced DNA damage.

**Conclusions:**

Taken together, this study shows that INI-43 pre-treatment significantly enhances cisplatin sensitivity in cervical cancer cells, mediated through stabilization of p53 and decreased nuclear import of NFκB. Hence this study suggests the possible synergistic use of nuclear import inhibition and cisplatin to treat cervical cancer.

**Supplementary Information:**

The online version contains supplementary material available at 10.1186/s12885-021-07819-3.

## Background

Multiple members of the nucleo-cytoplasmic transport system are deregulated in cancers and malignant tissues, including the importin protein Karyopherin Beta 1 (Kpnβ1) [1, 2]. Kpnβ1 is the major importing machinery in mammalian cells, which functions to traffic cargoes from the cytoplasm into the nucleus in interphase cells [3]. In the classical importing pathway, the nuclear localisation signal (NLS) bearing cargo is recognized and bound by the “adapter” protein – the alpha members of the Karyopherin family (Karyopherinα) [4]. The dimeric complex is then bound by Kpnβ1, which docks the newly formed trimeric complex to the nuclear pore complex (NPC). Interaction between Kpnβ1 and NPC components facilitates the transition of the transporter-cargo complex through the NPC [5]. Once on the nuclear side, RanGTP binds to the trimeric complex leading to its dissociation [6]. The cargo is freed to perform its nuclear function, while the Karyopherins are cycled back to the cytoplasm bound to RanGTP to perform the next round of nuclear import [7, 8]. During non-classical import, cargoes are imported in an adapter-independent manner and is shuttled directly by Kpnβ1 [9, 10]. Through shuttling a wide range of cargoes, Kpnβ1 regulates numerous cellular functions including inflammation, migration, apoptosis, morphology, circadian clock function and others [2, 11–13]. In addition to its nuclear importing function in interphase cells, Kpnβ1 also mediates cell division by regulating spindle assembly and mitotic exit [14, 15], thereby exhibiting pleiotropic functions in maintaining cell homeostasis and division.

Owing to its deregulation in multiple cancers, Kpnβ1 has been studied as a target for anti-cancer treatment. Multiple studies have shown that inhibition of Kpnβ1 exhibited broad-spectrum cancer killing activities through various mechanisms, including interfering with E2F1 activity [16], disruption of proteostasis [17], altering MET proto-oncogene expression and downregulating the epithelial-mesenchymal transition [18]. Most importantly, the impact of Kpnβ1 inhibition showed no toxicity on non-cancer cells, making Kpnβ1 an attractive target for cancer treatment [1]. Using an in vitro cervical cancer model, we previously demonstrated that siRNA mediated Kpnβ1 inhibition induced various mitotic defects, leading to a G2/M cell cycle arrest and ultimately apoptosis [19]. This further led to the in silico screening, and identification of the small molecule compound Inhibition of Nuclear Import-43 (INI-43), which exhibited nuclear-import inhibitory effect on Kpnβ1 cargoes and reduced cervical and oesophageal tumour growth in xenograft mouse models [13]. In addition, exogenous Kpnβ1 overexpression rescued the cytotoxic and nuclear import inhibitory effects of INI-43 on cancer cells, confirming that INI-43 exerts its impact via interfering with Kpnβ1 function [13].

In this study, we addressed the use of INI-43 in combination treatment (CT), by investigating its combined use with a clinically relevant chemotherapeutic agent – cisplatin. CT can be an effective way for treating cancer when participating agents engage in synergism, where the combined use produces greater anti-cancer effects compared to the additive effects of each when used individually. Successful combination chemotherapy translates into longer survival periods for cervical cancer patients, and this has been demonstrated for various combinations including topotecan, irinotecan, gemcitabine and docetaxel when paired with platinum based drugs [20–23]. More recently, various natural-derived compounds have been shown to synergize with cisplatin in treating cervical cancer, such as melatonin, epigallocatechin gallate, and genistein in vitro [24–26]. These findings suggest that platinum-based drugs hold great potential in combinational use. There is also evidence suggesting that interfering with the nuclear transport system could mediate sensitivity to chemotherapeutic agents. Kpnβ1 has been reported to confer docetaxel resistance, and siRNA mediated inhibition enhanced the cancer killing effect of docetaxel [27]. The combination of CRM1 inhibition and various conventional chemotherapeutic agents have also yielded promising results in reversing the chemo-resistance of many cancers [28–30], suggesting that manipulating nuclear transport may be a viable option in combination therapy. Selinexor, in particular, reduces the expression of DNA damage repair proteins and potentiates DNA damage-based therapy, including cisplatin [31].

Here we report that the combined use of nuclear import inhibitor INI-43 and cisplatin exhibited synergistic anti-cancer effects in cervical cancer cells. Furthermore, we show that enhanced cell death is mediated through p53 and NFκB function. The advantage of incorporating INI-43 into routine cisplatin use in treating cancer could be beneficial in two ways; firstly, to increase treatment response in patients exhibiting moderate resistance to cisplatin, and secondly, to achieve the same treatment outcome at lower doses of cisplatin, thereby minimizing undesired side effects associated with cisplatin.

## Methods

### Cell lines and tissue culture

HeLa, SiHa, CaSki and C33A cell lines were purchased from the American Type Culture Collection (ATCC) and maintained in Dulbecco’s Modified Eagle’s Medium (DMEM, Gibco, Life Technologies) containing 10% Fetal Bovine Serum (Gibco, Life Technologies), supplemented with 100 U/mL penicillin and 100 μg/mL streptomycin. Cells were cultured at 37 °C in a humidified chamber with 5% CO_2_. All cell lines were authenticated by DNA profiling using the Cell ID System (Promega, Madison, WI, USA).

### Half inhibitory concentration (IC_50_) determination

Cells were plated in 96-well plates and subjected to single or CT (2 h INI-43 pre-treatment followed by cisplatin treatment, without removing INI-43 from the media) for 48 h. Following treatment, MTT (Sigma) was added and 4 h later crystals solubilized using 10% SLS in 0.01 M HCl. Absorbencies were measured at 595 nm and IC_50_ values determined via plotting [Fa/(1-Fa)] in log scale against log cisplatin concentration, where *Fa* = $\frac{\mathsf{100}-\%\mathit{\mathsf{viable}\ \mathsf{cells}\ \mathsf{relative}\ \mathsf{to}\ \mathsf{the}\ \mathsf{untreated}}}{\mathsf{100}}$. The half inhibitory concentration was calculated using the formula *IC*_*50*_ *= 10*^*x-intercept*^*.*

### Drug washout experiments

Cells were plated in 96-well plates and subjected to 2 h INI-43 pre-treatment followed by cisplatin treatment, with or without removing INI-43 from the media, for 48 h. Following treatment, MTT (Sigma) was added and 4 h later crystals solubilized using 10% SLS in 0.01 M HCl.

### Caspase-3/7 assay

Cells were subjected to single or CT for 48 h, and caspase-3/7 activity monitored using the Promega Caspase-Glo^R^ 3/7 assay, according to the manufacturer’s instructions. Luminescence was measured using the Veritas™ microplate luminometer (Promega) and results standardized to viable cells in each treatment as determined by MTT assays performed in parallel.

### Combination index (CI) determination

To elucidate the nature of the combined use of INI-43 and cisplatin, the Chou-Talalay method was adopted [32]. Cell viability was determined after 48-h treatment at fixed INI-43 to cisplatin ratios of 1:3, 1:4 and 1:5 (Table S1). Cell viability was converted to fraction affected (Fa) and CI calculated using CompuSyn software (ComboSyn, Inc.).

### siRNA transfection

Cells were transfected using Transfectin (Bio-Rad) and 20 nM si-Kpnβ1 (H-7, sc-35,736, Santa Cruz) or 30 nM si-p53 (sc-29,435, Santa Cruz). Control cells were transfected with the equivalent amount of ctrl siRNA (si-ctrl, sc-37,007, Santa Cruz).

### Western blot analysis

For protein extraction, cells were washed with PBS and lysed using RIPA buffer (50 mM Tris-Cl, pH 7.4, 150 mM NaCl, 1% (w/v) sodium deoxycholate, 0.1% (v/v) SDS, 1% (v/v) Triton X-100, 2 mM EGTA, 2 mM EDTA, 50 mM NaF, 5 mM Na_2_P_2_O_7_, 1 X complete protease inhibitor cocktail (Roche) and 0.1 M Sodium Orthovanadate). For PARP cleavage analysis, dead cells were collected by centrifugation and combined with live cell lysates. Lysates were sonicated, centrifuged, and the supernatant quantified using the BCA Protein Assay Kit (Pierce, Thermo Scientific) according to the manufacturer’s instructions. Proteins were subjected to Western blot analysis using the following antibodies: rabbit anti-Kpnβ1 (H-300, sc-11,367, Santa Cruz), rabbit anti-β-tubulin (H-235, sc-9104, Santa Cruz), rabbit anti-PARP1/2 antibody (H-250, sc-7150, Santa Cruz), mouse anti-GAPDH (0411, sc-47,724, Santa Cruz), rabbit anti-p21 (H-164, sc-756, Santa Cruz), rabbit anti-Mcl-1 (H-260, sc-20,679, Santa Cruz), mouse anti-cyclin D1 (HD11, sc-246, Santa Cruz), rabbit anti-c-Myc (N-262, sc-764, Santa Cruz), mouse anti-p53 (DO-7, M7001, DakoCytomation), mouse anti-XIAP (610,763, BD Biosciences), and rabbit anti-phospho-Histone H2AX (γH2AX, Ser139, 20E3, #9718, Cell Signal).

### Nuclear/cytoplasmic fractionation

For nuclear/cytoplasmic protein extraction, cells were collected by trypsinization. Cell pellets were resuspended in 10 mM HEPES (pH 7.9), 50 mM NaCl, 0.5 M sucrose, 0.1 mM EDTA and 0.5% Triton X-100, followed by centrifugation at 1000 X G for 10 min to separate cytoplasmic (supernatant) and nuclear fractions (pellet). Cytoplasmic fractions were centrifuged at 14,000 X G for 15 min at 4 °C, and the supernatant stored at -80 °C. Nuclear pellets were washed in 10 mM HEPES, 10 mM KCl, 0.1 mM EDTA and 0.1 mM EGTA, and centrifuged at 1000 X G for 5 min. Pellets were then resuspended in 10 mM HEPES (pH 7.9), 500 mM NaCl, 0.1 mM EDTA, 0.1 mM EGTA and 1% (v/v) NP-40 and vortexed for 15 min at 4 °C to extract the nuclear protein, followed by centrifugation at 14,000 X G for 10 min. The fractions were quantified using the BCA Protein Assay Kit (Pierce, Thermo Scientific) according to the manufacturer’s instructions, followed by western blot analysis.

### p53 half-life (T_1/2_) determination

Cells were treated with 5 μM INI-43 or DMSO for 2 hours or transfected with 20 nM si-ctrl or si-Kpnβ1 for 48 h prior to p53 half-life determination. Cells were treated with 50 μg/mL cycloheximide (CHX), and protein harvested at 0, 15, 30, 45, 60 and 90 min after CHX treatment. p53 content was analysed by western blotting. Bands were quantified by densitometrical scanning using ImageJ, normalised to GAPDH and expressed as a value relative to p53 intensity at time 0. Relative band intensities were plotted in log scale against time of CHX treatment and a linear trendline drawn. The half-life was calculated using the formula T_1/2_ (minutes) = Log(2)/[slope].

### Immunofluorescence

SiHa cells were plated on glass coverslips and treated for 24 h before fixation with 4% paraformaldehyde. Cells were permeabilised using 0.5% Triton-X-100/PBS and blocked using 1% BSA/PBST with 0.3 M Glycine. Primary antibody incubations were performed in 1% BSA/PBST, followed by secondary antibody incubation (Cy3 conjugated goat anti-rabbit, Jackson ImmunoResearch). Cell nuclei were counterstained with 0.5 μg/mL DAPI, and images captured using the Zeiss inverted fluorescence microscope under 100 X oil immersion.

### Luciferase reporter assay

SiHa cells were transfected with 100 ng p65-luciferase reporter construct (containing five copies of the p65-binding site, Promega) or 200 ng p53-luciferase reporter construct (containing thirteen wildtype p53 binding sites, Addgene plasmid #16442, Addgene Plasmid Repository [33] and 10 ng pRL-TK (encoding Renilla luciferase, Promega), using Genecellin transfection reagent (Celtic Molecular Diagnostics). The following day cells were treated with 5 μM INI-43 for 2 h, followed by 30 μM cisplatin for 24 h, and luciferase activity assayed using the Dual-Luciferase Report assay system (Promega), according to the manufacturer’s instructions. Luciferase readings were measured using the Veritas™ microplate luminometer (Promega) and normalised to Renilla luciferase from the same extract.

### Statistical analysis

For all data comparisons, the Student’s t test was performed using Microsoft Excel. A *p* value of < 0.05 was considered statistically significant.

## Results

### INI-43 pre-treatment enhanced HeLa and SiHa cell sensitivity to cisplatin

**Fig. 1:**
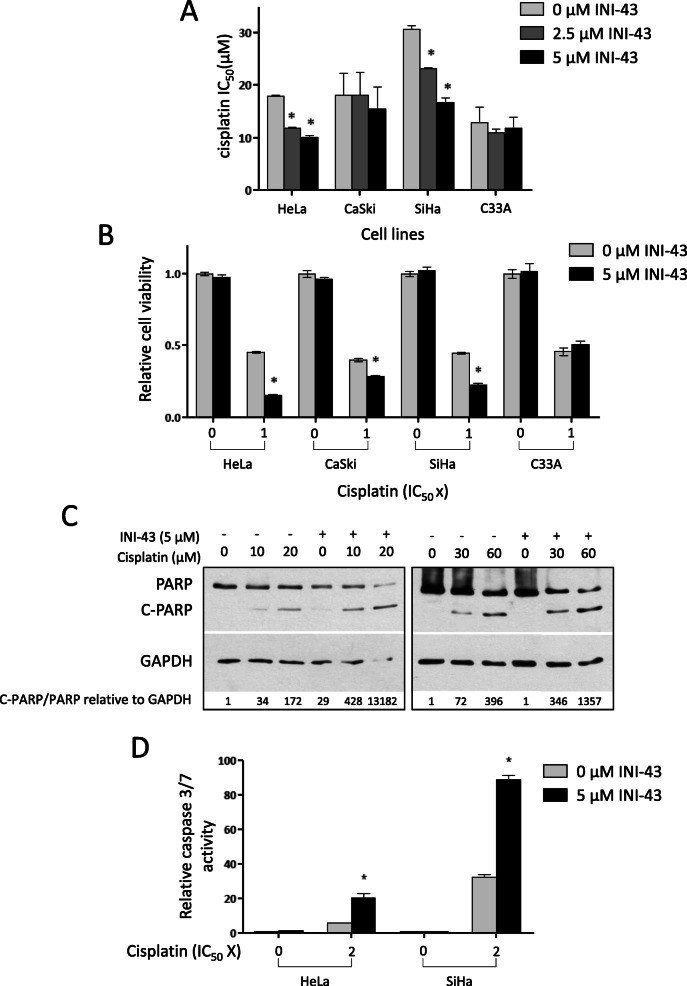
INI-43 pre-treatment significantly enhances cisplatin sensitivity in cervical cancer cell lines HeLa and SiHa. **a** Cisplatin IC_50_ values in cervical cancer cell lines HeLa, CaSki, SiHa and C33A pre-treated with 2.5 μM and 5 μM INI-43 for 2 h, compared to control cells receiving no pre-treatment. Results shown are the mean IC_50_ value ± SEM of three independent experiments (*n* = 6). **b** MTT cell proliferation assay 48 h post-treatment, showing increased cisplatin sensitivity in HeLa, CaSki and SiHa cells after pre-treatment with 5 μM INI-43. **c** Western blot analysis showing enhanced PARP cleavage in INI-43 and cisplatin combination treated HeLa and SiHa cells. GAPDH was used as a loading control, and quantification via densitometry is shown. The full-length blots are shown in Supplementary Fig. 2. **d** Caspase-3/7 activity in HeLa and SiHa cells was significantly enhanced upon INI-43 and cisplatin combination treatment, compared to cisplatin single treatment. In all cases, results shown are the mean ± SEM of experiments performed in triplicate and repeated three independent times (*p < 0.05)

To investigate whether nuclear import inhibition could influence cancer cell sensitivity to cisplatin treatment, cisplatin IC_50_ values were compared between cervical cancer cells with and without INI-43 pre-treatment. Pre-treatment was conducted at sublethal INI-43 concentrations (≤10 μM) for 2 h (concentrations which were previously shown to reduce nuclear import of various Kpnβ1 cargoes [13]), followed by cisplatin treatment. Cisplatin IC_50_ values after 48-h treatments were 18.0 μM, 18.1 μM, 30.8 μM and 12.8 μM for HeLa, CaSki, SiHa and C33A, respectively. However, when cells were pre-treated with INI-43, a significant dose-dependent decrease in cisplatin IC_50_ was observed in both HeLa and SiHa cells (44 and 46% in HeLa and SiHa cells, respectively) (Fig. 1a). A small reduction in cisplatin IC_50_ was observed in CaSki cells and no change in cisplatin IC_50_ observed in C33A cells.

Cell viability was next examined at fixed cisplatin concentrations, with or without INI-43 pre-treatment. Figure 1b shows that in HeLa, CaSki and SiHa cells, CT resulted in significantly decreased cell viability compared to their cisplatin-only treated counterparts. In line with the cisplatin IC_50_ results, C33A showed no change in cell viability after single or CT. As 5 μM INI-43 on its own did not affect cell viability across all cell lines, this suggests that the enhanced cell death observed in the CT was due to the combined action of INI-43 and cisplatin, rather than addition of independent effects of the two drugs.

Since INI-43 was not removed from the cells before cisplatin treatment it was next determined whether the effects of INI-43 would be sustained following drug removal, or whether the INI-43 treatment effects were transient. Washout experiments were performed where cells were incubated with INI-43 for 2 h, and thereafter either treated with cisplatin (with INI-43 still present), treated with cisplatin after INI-43 removal (washout 1), or treated with cisplatin 2 h after INI-43 removal (washout 2). Results showed that even after INI-43 was removed before cisplatin treatment there was still significantly reduced cell viability in response to the combination treatment when compared to the effects of cisplatin alone, suggesting that the effects of INI-43 are not reversible following drug washout (Supplementary Fig. 1). The enhancement of cell death upon CT was slightly reduced in HeLa cells after INI-43 washout, but this is likely due to the rapid doubling time of HeLa cells, and thus quick synthesis of nascent Kpnβ1 which would begin to counteract the effects of INI-43 over time.

To determine whether INI-43-cisplatin CT resulted in increased apoptosis, PARP cleavage and caspase-3/7 activation were assayed. Protein from live and dead cells was collected and PARP status examined by western blot. In both HeLa and SiHa cells, enhanced PARP cleavage was observed in the combination treated cells compared to those receiving cisplatin only (Fig. 1c). Supporting the cell viability data, 5 μM INI-43 treatment on its own showed negligible apoptosis. Investigation of caspase-3/7 activation revealed that combination treated cells exhibited increased caspase-3/7 activation compared to cisplatin only treated cells (3.6-fold and 2.8-fold increase in HeLa cells and SiHa cells, respectively) (Fig. 1d). These results suggest that nuclear import inhibition via INI-43 pre-treatment sensitized both HeLa and SiHa cells to cisplatin through enhanced activation of apoptosis.

### INI-43 and cisplatin combination treatment resulted in synergistically enhanced cell death

**Fig. 2:**
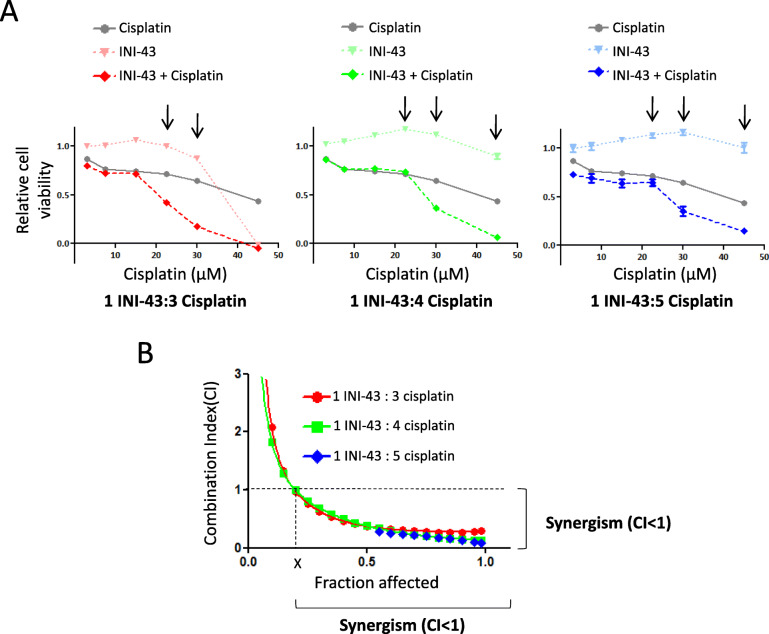
Combination index (CI) evaluation shows that INI-43 and cisplatin combination treatment results in a synergistic anti-cancer effect in SiHa cells. **a** Cells were subjected to INI-43, cisplatin or the combination treatment for 48 h. Combination treatments were carried out at fixed INI-43-to-cisplatin ratios of 1:3, 1:4 and 1:5 (see Table S1). Viable cells were determined using the MTT assay and expressed relative to untreated. Arrows indicate enhanced cell death. **b** CI values were calculated using CompuSyn software and plotted against the fraction affected. Data shown are the mean ± SEM (*n* = 5) and experiments were repeated at least two independent times

Since the concentration of INI-43 used was not sufficient to induce significant cell death alone, and yet in combination with cisplatin it significantly enhanced cell death, it was proposed that INI-43 and cisplatin engaged in a synergistic interaction, where the cytotoxic effect of their combined use was greater than the additive effects of either drug used independently. To test this, the combination index (CI) was evaluated, according to the Chou-Talalay method, using a fixed dose ratio [32]. SiHa cells were treated with INI-43 and cisplatin at varying concentrations to give INI-43-to-cisplatin ratios of 1:3, 1:4 or 1:5 (Table S1). Cells were pre-incubated with respective INI-43 concentrations for 2 h prior to cisplatin treatment. Results showed that while cisplatin reduced cell viability in a dose-dependent manner, pre-treatment with INI-43 significantly enhanced this effect (Fig. 2a). Based on the cell viability results, the CI values were calculated using CompuSyn software (ComboSyn, Inc.) and plotted against Fraction Affected (Fa), where Fa = 0 and Fa = 1 equates to no cell death and complete cell death, respectively. At Fa > 0.2, CI values were below 1 for INI-43 to cisplatin ratios of 1:3, 1:4 and 1:5, revealing synergistically enhanced cell death (Fig. 2b).

### Kpnβ1 knock-down sensitized cervical cancer cells to cisplatin

**Fig. 3:**
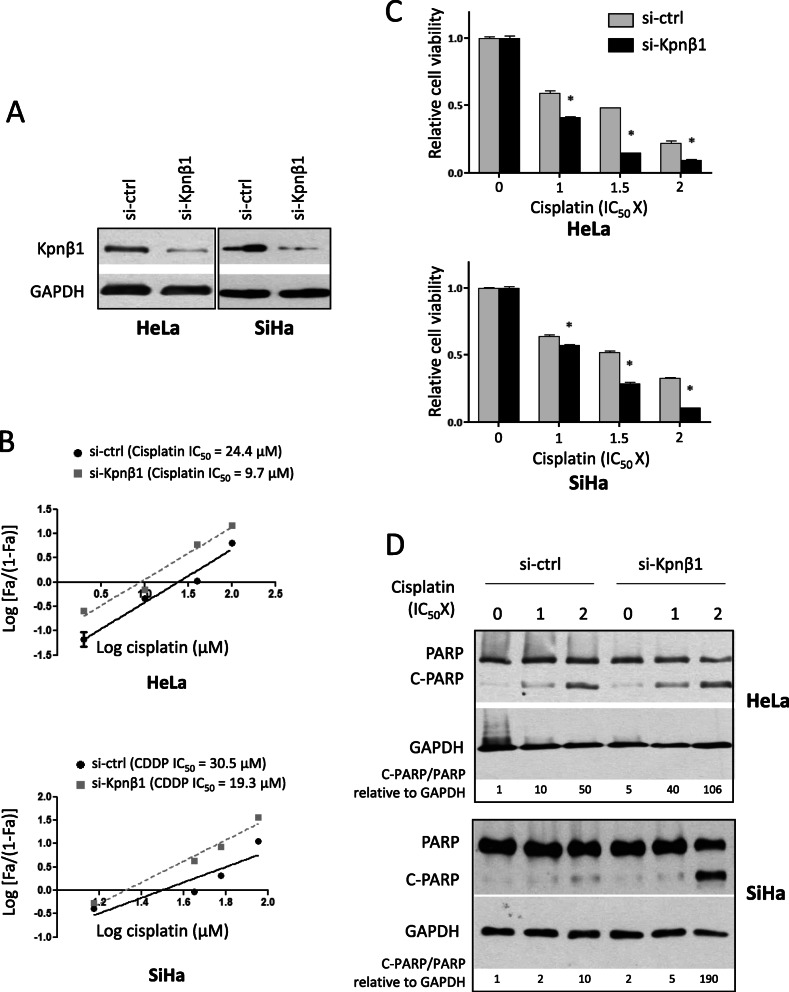
Kpnβ1 knock-down enhances cisplatin sensitivity in cervical cancer cells. **a** HeLa and SiHa cells were transfected with siRNA for 48 h, after which Kpnβ1 knock-down was confirmed by western blotting, with GAPDH as the loading control. The full-length blots are shown in Supplementary Fig. 3A. **b** Cisplatin IC_50_ values were determined in Kpnβ1 knock-down HeLa and SiHa cells and results showed a decrease in cisplatin IC_50_ in both cell lines when transfected with si-Kpnβ1 compared to si-ctrl. Data shown are results ± SEM (n = 6) of a representative experiment performed two times. **c** Kpnβ1 knock-down affected cell viability in response to cisplatin treatment in HeLa and SiHa cells. Control and Kpnβ1 knock-down cells were treated with cisplatin for 48 h before viable cells were measured using the MTT assay. Data shown are mean ± SEM (n = 6) of one representative experiment repeated two times (*p < 0.05). **d** Western blot showing that Kpnβ1 knock-down increased PARP cleavage after cisplatin treatment in HeLa and SiHa cells. GAPDH was included as a loading control, and densitometrical quantification of C-PARP/PARP relative to GAPDH is shown. The full-length blots are shown in Supplementary Fig. 2B. Results are representative of experiments performed two independent times

To confirm that the enhancing effect of INI-43 on cisplatin cytotoxicity was due specifically to nuclear import inhibition, rather than off-target effects, cisplatin sensitivity was examined in Kpnβ1 knock-down cells. Cells were transfected with Kpnβ1 targeting siRNA (si-Kpnβ1) or control siRNA (si-ctrl), and cisplatin sensitivity determined. Successful Kpnβ1 knock-down was confirmed by western blotting 48 h post transfection, at which point cisplatin treatment began (Fig. 3a). In Kpnβ1 knock-down cells, there was a significant reduction of cisplatin IC_50_ from 24.4 μM in the control cells to 9.7 μM in HeLa cells, and 30.5 μM in the control cells to 19.3 μM in SiHa cells (Fig. 3b). To confirm this effect, cell viability was measured after cisplatin treatment in Kpnβ1 knock-down and control siRNA transfected cells. To eliminate the cell death that was caused by Kpnβ1 knock-down, cell viability was normalized to untreated cells in each transfection group. Results indicated that Kpnβ1 knock-down cells were more sensitive to cisplatin-induced cell death at all concentrations tested (Fig. 3c). Furthermore, Kpnβ1 knock-down HeLa and SiHa cells exhibited visibly increased PARP cleavage after cisplatin treatment compared to control siRNA-transfected cisplatin-treated cells (Fig. 3d). Collectively, these results show that Kpnβ1 knock-down enhanced sensitivity to cisplatin, similarly to that observed after INI-43 treatment, supporting that INI-43 increases cisplatin sensitivity by disrupting Kpnβ1 function.

### p53 is an important mediator of INI-43-cisplatin-induced cell death

**Fig. 4:**
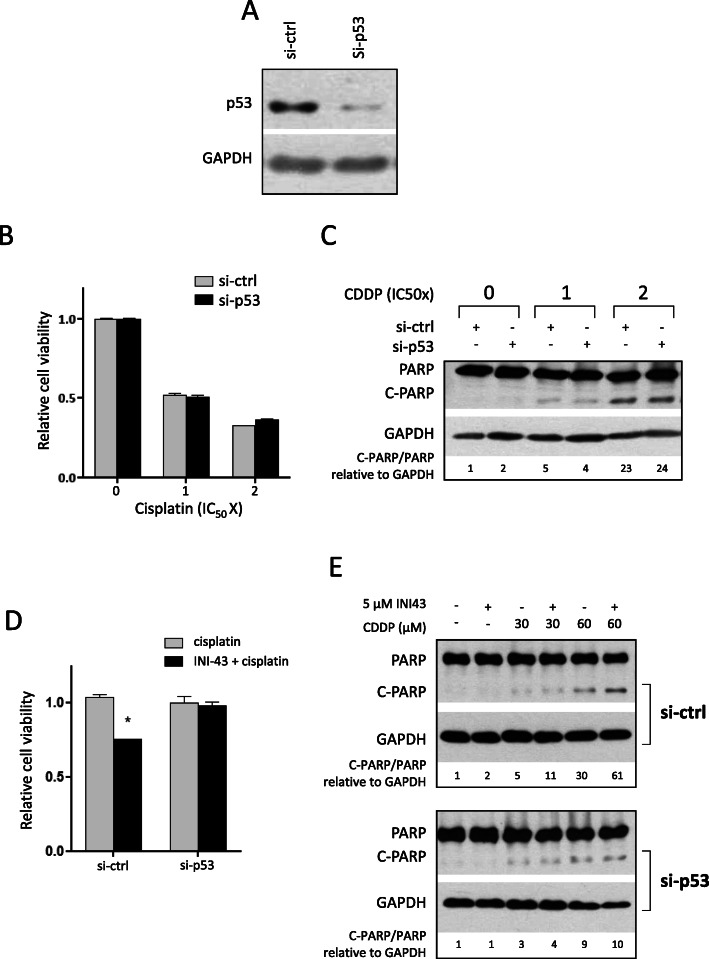
p53 inhibition does not affect cisplatin sensitivity but is required for the enhanced cell death observed in the combination treatment. **a** SiHa cells were transfected with siRNA for 48 h and p53 knock-down confirmed via western blot. GAPDH was included as a loading control. The full-length blots are shown in Supplementary Fig. 4A. **b** MTT assay showing that p53 knock-down does not affect cell viability after 48-h cisplatin treatment. Data shown are the mean ± SEM of experiments performed in triplicate and repeated two independent times. **c** Western blot showing that p53 knock-down does not affect cisplatin-induced PARP cleavage in SiHa cells. GAPDH was included as loading control, and densitometrical quantification is presented. Results shown are representative of experiments performed three times. The full-length blots are shown in Supplementary Fig. 4B. **d** MTT assay comparing cell viability between single cisplatin treatment and INI-43-cisplatin combination treatment, in p53 knock-down SiHa cells. To compare the degree of enhancement of cell death as a result of the combination treatment, cell viability was normalized to cells receiving single cisplatin treatment. Results shown are mean ± SEM of experiments performed in triplicate and repeated two independent times (*p < 0.05). **e** Western blot showing that p53 knock-down abrogated the enhancement of PARP cleavage observed after combination treatment compared to single cisplatin treatment. GAPDH was included as the loading control, and densitometrical quantification is shown. The full-length blots are shown in Supplementary Fig. 4C. Results shown are representative of three independent experiments

To elucidate whether p53 might play a role in the cellular response to cisplatin and furthermore, whether the enhanced cisplatin sensitivity in INI-43 pre-treated cells involved p53, the effects of p53 knock-down were examined. p53 knock-down was confirmed via western blot 48 h post transfection, at which point cells were subjected to drug treatments as previously described (Fig. 4a). After single cisplatin treatment, p53 knock-down cells exhibited similar cell viability to si-ctrl transfected cells, suggesting that p53 was not involved in cisplatin-induced cell death (Fig. 4b). These results were validated by PARP cleavage analysis, where similar levels of cleaved PARP were observed between the control and p53 knock-down cells at the same cisplatin concentrations (Fig. 4c).

The impact of p53 on the enhancement of cell death observed after INI-43 and cisplatin CT was next examined. To quantify the “additional” cell death associated with the CT, cell viability was normalized to single cisplatin treatment. As previously established, a significant reduction in cell viability was observed after INI-43 and cisplatin CT, compared to single cisplatin treatment in the si-ctrl transfected cells. However, p53 knock-down cells exhibited similar sensitivity to single and CT, i.e., INI-43 pre-treatment induced sensitisation to cisplatin was lost with p53 inhibition (Fig. 4d). Examination of PARP cleavage in these cells showed similar results; while si-ctrl transfected cells showed increased levels of cleaved PARP after CT compared to single cisplatin treatment, p53 knock-down cells exhibited similar levels of cleaved PARP between single and CT (Fig. 4e). These results showed that cisplatin alone induced cell death is p53-independent, however, p53 appears to be critical for the enhancement of cell death observed in the CT, as p53 knock-down abrogated this effect.

### INI-43 pre-treatment stabilized p53 via Kpnβ1 inhibition

**Fig. 5:**
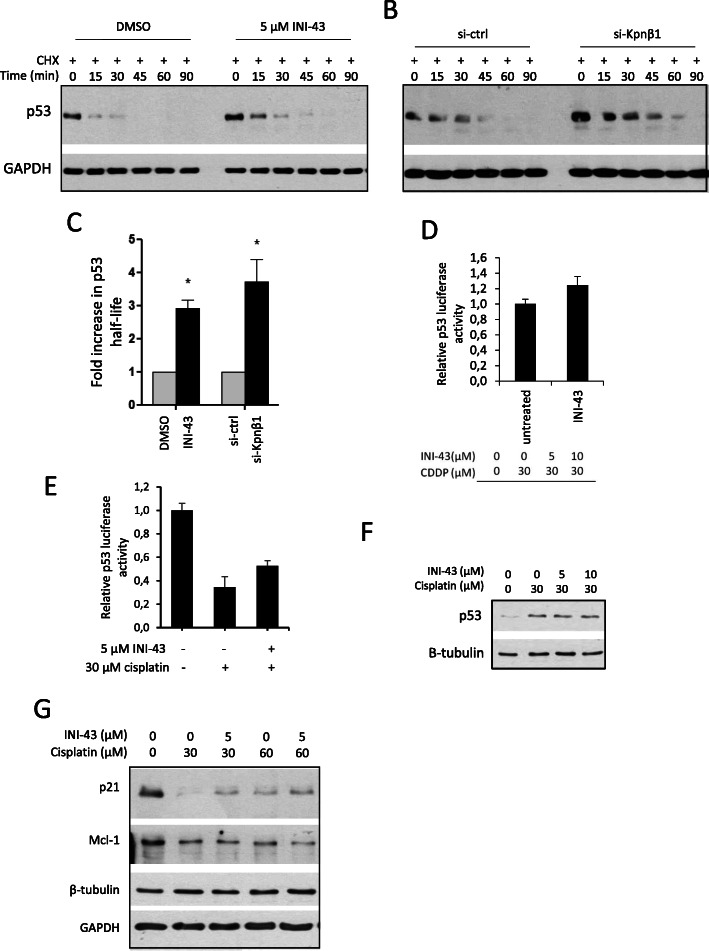
Inhibition of Kpnβ1 results in increased p53 stability and reporter activity, as well as increased p21 and decreased Mcl-1 in response to cisplatin. **a**, **b** SiHa cells were treated with DMSO or 5 μM INI-43 for 2 h (**a**) or transfected with si-ctrl or si-Kpnβ1 for 48 h (**b**) followed by 50 μg/mL CHX treatment. Protein was harvested at the indicated time points, and p53 content analyzed by western blot. GAPDH served as the loading control. The full-length blots are shown in Supplementary Fig. 5A and 5B. **c** The fold increase in p53 half-life is shown as the mean ± SEM from the three independent experiments (*p < 0.05). **d** p53 reporter activity is increased upon 24 h 5 μM INI-43 treatment of SiHa cells (*p < 0.05). Experiments were performed in triplicate and repeated at least three independent times. **e** p53 reporter activity is enhanced in INI-43-cisplatin combination treated SiHa cells, compared to cells treated with cisplatin alone (*p < 0.05). Experiments were performed in triplicate and repeated at least three independent times. **f** Western blot showing levels of p53 after single and combination treatment. β-tubulin served as a loading control. The full-length blots are shown in Supplementary Fig. 5C. **g** Western blot showing levels of p53 targets p21 and Mcl-1 after single and combination treatment. β-tubulin served as a loading control for Mcl-1 and GAPDH for p21. Results shown are representative of two independent experiments. The full-length blots are shown in Supplementary Fig. 5D

p53 is known to be highly unstable in HPV positive cells due to the activity of HPV oncoprotein E6 [34], and as SiHa is an HPV 16 positive cell line known to express E6 [35], it was possible that INI-43 treatment interfered with p53 stability, thereby altering cell sensitivity to cisplatin treatment. To test this, the rate of p53 degradation was monitored in cyclohexmide (CHX)-treated cells. Cells were treated with 5 μM INI-43 or DMSO for 2 h, whereafter CHX was added and protein extracted at various time points after CHX treatment. Western blot analysis showed an increase in p53 stability in INI-43 treated cells compared to DMSO treated control cells (Fig. 5a). To confirm that the prolonged p53 presence observed after INI-43 treatment was associated with Kpnβ1 inhibition, p53 levels were also examined in Kpnβ1 knock-down cells after CHX treatment. Similar to that observed after INI-43 treatment, Kpnβ1 knock-down cells were able to sustain p53 for a longer period after CHX treatment (Fig. 5b). The half-life of p53 was calculated and an approximate 2.9-fold and 3.7-fold increase in half-life was observed, in INI-43 treated and si-Kpnβ1 transfected cells, respectively, compared to control cells (Fig. 5c). Similar observations were made in HeLa cells, where Kpnβ1 knock-down increased p53 half-life by approximately 3.3-fold (data not shown). To investigate whether the stabilization of p53 had any functional relevance, p53 reporter activity was measured after INI-43 treatment. 5 μM INI-43 treatment led to a small but significant increase in p53 activity, consistent with its prolonged half-life (Fig. 5d).

To relate these findings to combination treated cells, p53 reporter activity was measured in SiHa cells treated with INI-43 and cisplatin, compared to single cisplatin treatment. Interestingly, p53 reporter activity was significantly reduced upon single cisplatin treatment, in line with the lack of involvement of p53 in cisplatin-induced cell death observed in Fig. 4 (Fig. 5e). However, with INI-43 pre-treatment, p53 reporter activity was significantly increased (Fig. 5e). p53 protein levels, on the other hand, were increased after single cisplatin treatment and after treatment with INI-43 and cisplatin (Fig. 5f). These results show that while p53 is stabilized following treatment with cisplatin, its activity is inhibited. Pre-treatment with INI-43, however, results in increased p53 activity. Following from the increased p53 reporter activity, the levels of two proteins known to be regulated by p53 were investigated: p21 which is positively regulated by p53, and Mcl-1 which is transcriptionally repressed by p53. Western blot analysis showed that cisplatin treatment at 30 μM and 60 μM decreased levels of both p21 and Mcl-1. However, in cells receiving both INI-43 and cisplatin, p21 levels were elevated compared to single cisplatin treatment at both 30 and 60 μM concentrations, and Mcl-1 levels were reduced at 60 μM cisplatin (Fig. 5g). These results confirm the involvement of p53 and p53 downstream targets in the INI-43-mediated enhanced cytotoxicity to cisplatin.

### INI-43-cisplatin combination treatment reduced cisplatin-induced nuclear accumulation of NFκB

**Fig. 6:**
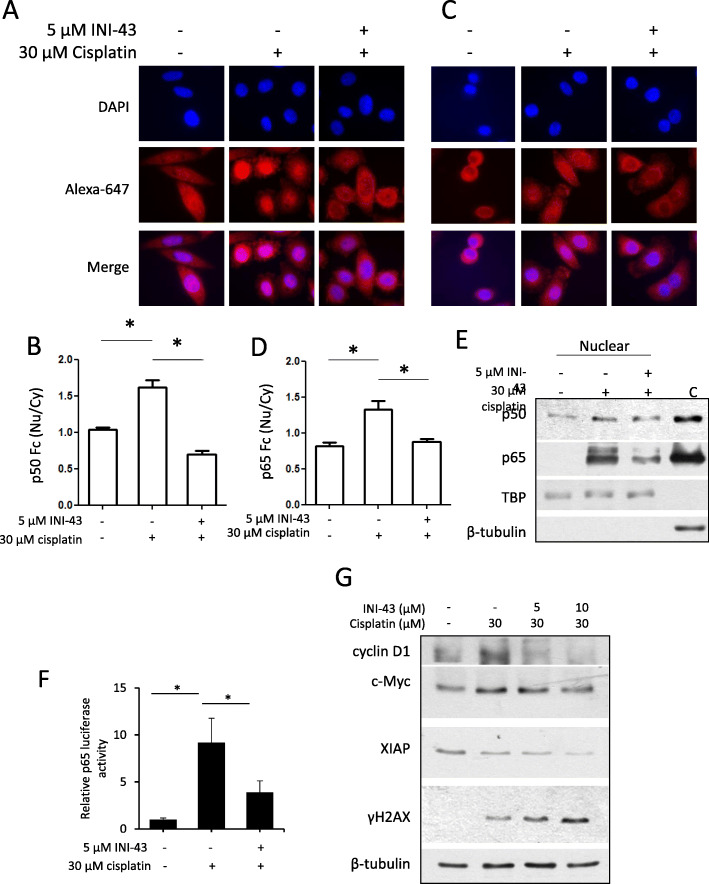
INI-43 pre-treatment reduces the nuclear localization of NFκB and expression of its targets and enhances DNA damage after cisplatin treatment in SiHa cells. **a**, **c** Distribution of NFκB subunits p50 (**a**) and p65 (**c**) were analyzed by immunofluorescence after single (30 μM cisplatin) or combination treatment. **b**, **d** Fluorescence intensities were quantified using ImageJ and expressed as nuclear fluorescence relative to cytoplasmic fluorescence (Fc (Nu/Cy)) for p50 (**b**) and p65 (**d**). Results shown are representative images for each condition (**a**, **c**), and mean ± SEM of 6 cells (*p < 0.05, **b**, **d**). **e** Western blot analysis showing increased nuclear p50 and p65 levels after cisplatin treatment, which was reduced if cells were pre-treated with INI-43. TBP served as the nuclear loading control and β-tubulin to confirm that pure nuclear lysates were obtained in comparison to a random cytoplasmic protein sample ‘C’. Results shown are representative of experiments conducted three independent times. The full-length blots are shown in Supplementary Fig. 6A. **f** p65 reporter assay showing increased p65 luciferase activity after 30 μM single cisplatin treatment, which was reduced with INI-43 pre-treatment (*p < 0.05). **g** Western blot showing changing levels of various NFκB targets (cyclin D1, c-Myc and XIAP) after single or combination treatment, and enhanced phosphorylation of H2A.X (γH2AX) in combination treated cells, indicative of increased DNA damage. β-tubulin was included as the loading control, and results shown are representative of experiments performed at least two independent times. The full-length blots are shown in Supplementary Fig. 6B

We have previously shown that treating cancer cells with INI-43 prohibited PMA-stimulated nuclear entry of NFκB-p65 [13]. Others have reported that in SiHa cells, cisplatin treatment leads to activation of NFκB which contributes to cisplatin resistance in various cancer models [36]. As NFκB activation requires nuclear translocation to initiate transcription of downstream targets, NFκB nuclear localization was evaluated by immunofluorescence after single and CT, as an indication of activity. Results showed that while cisplatin treatment stimulated nuclear localization of NFκB-p50 and NFκB-p65, INI-43 pre-treatment prevented this nuclear translocation of both NFκB subunits upon cisplatin treatment (Fig. 6a and c). Fluorescence quantification supported these results, where cisplatin treatment led to a significant increase in nuclear fluorescence relative to cytoplasmic fluorescence (Fc (Nu/Cy)), and the INI-43-cisplatin CT significantly reduced this effect (Fig. 6b and d).

To independently confirm these results, nuclear and cytoplasmic protein fractions were isolated from cisplatin-treated or combination treated SiHa cells. Western blot analysis showed that cisplatin treatment resulted in increased levels of both NFκB-p50 and NFκB-p65 in the nucleus, and that INI-43-cisplatin CT reduced this effect (Fig. 6e). Next, it was determined whether the altered localization of NFκB-p50 and NFκB-p65 after CT translated into functional significance. p65 reporter activity was measured after single or CT, and results showed that p65 reporter activity was induced upon cisplatin treatment, but the increase in p65 activity was reduced when cells were pre-treated with INI-43 (Fig. 6f). The expression of three downstream targets of NFκB known to respond to cisplatin treatment were hence examined, namely cyclin D1, c-Myc and X Chromosome Linked Inhibitor of Apoptosis (XIAP) [37–39]. Western blot analysis showed that single cisplatin treatment led to elevated levels of cyclin D1 and c-Myc (Fig. 6g). Moreover, the levels of cyclin D1, c-Myc and XIAP were all reduced in INI-43-cisplatin combination treated cells in a concentration dependent manner compared to single cisplatin treated cells (Fig. 6g). As both cyclin D1 and c-Myc have been shown to confer chemoresistance via increasing the cells’ DNA repair capacity [40, 41], we examined whether their decreased levels observed in the CT had an impact on cisplatin-induced DNA damage. The level of phosphorylated Histone 2AX (γH2AX), a marker for DNA damage was examined 24 h after single or CT by western blot. Results showed that the INI-43-cisplatin CT increased γH2AX levels in a concentration dependent manner, suggesting that pre-treating cells with INI-43 prior to cisplatin treatment enhanced the DNA damaging effect of cisplatin (Fig. 6g).

Together, these results demonstrate that INI-43 pre-treatment effectively reduced nuclear accumulation and activity of NFκB, resulting in decreased expression of cyclin D1, c-Myc and XIAP, and impaired DNA repair ability, sensitising the cells to cisplatin treatment.

## Discussion

This study is a first to demonstrate that inhibition of Kpnβ1 is an effective way to enhance the anti-cancer effects of cisplatin, and that both cisplatin sensitive, HeLa, and the more resistant, SiHa cervical cancer cells were responsive to this treatment. Furthermore, combination index analysis indicated a synergistic interaction between INI-43 and cisplatin, where their combined use produced greater anti-cancer effects compared to the added effects when used alone.

To understand the mechanism of action underlying the increased cisplatin sensitivity observed in the CT, proteins involved in cisplatin response were investigated, including both p53 and NFκB. Whilst p53 is widely accepted as a tumour suppressor protein important in guarding the genome and regulating apoptosis, some evidence has emerged to demonstrate that p53 can also promote oncogenesis by preventing apoptosis [42], suggesting that p53 can be involved in cisplatin resistance or cisplatin-induced apoptosis. p53 knock-down experiments demonstrated that p53 is involved in the pro-apoptotic pathway in our model system, but only in response to the CT, as p53 knock-down did not affect sensitivity to single cisplatin treatment.

SiHa cells are HPV positive, harbouring the HPV16 E6 oncoprotein [35], which has been reported to directly associate with p53 and induce its degradation [34]. This results in a highly unstable p53, which is supported by our observation whereby p53 is rapidly degraded after CHX treatment. We observed stabilisation of p53 in response to INI-43 treatment and Kpnβ1 knock-down in SiHa cells. Stabilization of p53 has also been observed in HPV16 and HPV18 positive Kpnβ1 knock-down CaSki cells [19]. The stabilisation of p53 upon Kpnβ1 inhibition is likely due to the role of Kpnβ1 in mediating p53 and HPV E6 nuclear entry. p53 is reported to enter the nucleus via Kpnα4 (Importin α3) and Kpnβ1 [43, 44], however, it is known that there is redundancy between nuclear transport receptors, and we have previously shown an accumulation of p53 in the nucleus and cytoplasm upon Kpnβ1 inhibition [19], suggesting p53 still has access to the nucleus when Kpnβ1 is inhibited. HPV E6 protein has also been reported to enter the nucleus via Kpnβ1 and Kpnβ2 [45]. It is possible that Kpnβ1 inhibition with INI-43 affects nuclear entry of p53 and HPV E6 to varying extents, interfering with HPV E6-mediated p53 degradation, and resulting in p53 stabilisation. The exact mechanism involved, however, requires further investigation. Interestingly, inhibition of CRM1 via small molecules KPT-185 and leptomycin B has also been shown to stabilize p53 in other cancers [46, 47]. Together with our findings, these data suggest that interfering with the nuclear transport system in either directions has stabilizing effects on p53.

In combination treated cells, there was increased p53 activity after INI-43 pre-treatment, which associated with increased responsiveness to cisplatin treatment. We propose that in our model system, p53 protein accumulates upon cisplatin treatment, however, the action of HPV E6 renders it inactive [48]. p53 knock-down thus had little effect on cisplatin induced cell death. However, in the combination treated cells it is possible that the inhibition of Kpnβ1 interferes with p53 and HPV E6 nuclear entry, altering the levels of E6-bound p53 in the nucleus, and the p53 that accumulates is more readily available for apoptotic induction when cells are challenged with cisplatin. This could also explain why INI-43 did not sensitize C33A cells to cisplatin, as C33A cells are HPV negative and carry a non-functional mutant p53 [49].

In addition to enhanced p53 stability and reporter activity, increased p21 levels and decreased Mcl-1 levels were observed in INI-43 pre-treated cells compared to non-pre-treated cells in response to cisplatin treatment. p53 is known to positively regulate p21 expression and repress Mcl-1 [50, 51]. Furthermore, the elevated caspase-3/7 activity observed in the CT could be associated with the decreased levels of Mcl-1, as Mcl-1 is known to promote survival by inhibiting events preceding mitochondrial release of cytochrome C [52]. Whilst the link between Kpnβ1 inhibition and p53 stabilization is demonstrated in our results, further experiments should be performed to address how nuclear import inhibition leads to p53 stabilization, and whether this is mediated through interfering with HPV 16 E6 activity.

Interestingly, with opposing roles in apoptosis, NFκB and p53 have been shown to mutually antagonize the transcriptional activity of each other [53], and our results showed there was also a differential distribution of NFκB subunits p50 and p65 in cells receiving the single cisplatin and CT. NFκB is an important response factor to stress signals, including cisplatin-induced DNA damage [54], whereupon it relocates to the nucleus to promote the transcription of various genes involved in DNA repair and survival [36]. As NFκB is reliant on Kpnβ1/Karyopherinα for nuclear entry [55], the localisation of NFκB was measured after INI-43 treatment which showed that INI-43 inhibited cisplatin-induced nuclear import of NFκB, as well as the expression of its transcriptional targets cyclin D1, c-Myc and XIAP. This coincided with elevated levels of γH2AX, suggesting that Kpnβ1 inhibition either augmented the DNA damaging capacity of cisplatin, or, alternatively, impaired the DNA repair response. c-Myc confers chemoresistance via suppressing BIN1, an inhibitor of PARP-1 involved in DNA repair activity, thereby increasing tolerance to DNA damage and conferring cisplatin resistance [41]. XIAP promotes survival by directly binding to and inhibiting the activities of caspase-3, caspase-7 and possibly caspase-9 [56]. Cyclin D1, best known for driving cell cycle from G1 to S phase, is also involved in DNA damage repair in association with Rad51 [57], and its inhibition impairs DNA repair capacity leading to sensitization of cancer cells to cisplatin [40]. Our results showed that INI-43-cisplatin CT results in reduced levels of these DNA-repair and anti-apoptotic proteins, possibly via decreasing NFκB nuclear import and transcriptional activity. However, it must also be noted that the response of these proteins to INI-43-cisplatin CT may also be attributed to other mechanisms besides NFκB. For example, Yang et al. (2019) recently showed that in addition to blocking NFκB nuclear translocation, Kpnβ1 inhibition also reduced the nuclear translocation of c-Myc in prostate cancer cells [58].

It is worth noting that a previous study from our group demonstrated that Kpnβ1 overexpression similarly sensitized cervical cancer cells to cisplatin. Although this may seem contradictory to the current study, it is important to know that overexpression of Kpnβ1 (above what is already expressed in the cancer cells) did not benefit cancer cell survival. Rather, it reduced cancerous properties including reduced cell proliferation, increased cell adhesion and mesenchymal-to-epithelial transition [11]. Hence, it appears that it is a tightly controlled balance of Kpnβ1 level that is beneficial to the cancerous traits, and that perturbation of this equilibrium in either direction (overexpression or inhibition) is detrimental to the survivability of cancer cells. This is indeed, supported by earlier works which demonstrated that Kpnβ1 overexpression led to mitotic catastrophes, which was avoided by co-overexpressing other Kpnβ1 interacting partners [14, 59, 60]. While this phenomenon is interesting, inhibition of Kpnβ1 may be a more viable strategy as a therapeutic option and hence was pursued in the current study in combination with cisplatin.

## Conclusions

Taken together, this study shows that Kpnβ1 inhibition sensitizes cervical cancer cells to cisplatin, suggesting that coupling nuclear import inhibition with cisplatin may be an effective anti-cancer approach. This is mediated through stabilisation of p53 and prevention of NKκB nuclear localization, leading to alterations in the expression of various downstream targets such as XIAP, c-Myc, and Mcl-1. These proteins are known to confer cisplatin resistance in a variety of cancers, and their inhibition through genetic or pharmacological approaches have been demonstrated to increase sensitivity to chemotherapeutic agents [39, 61, 62]. The abrogation of enhanced cell death in combination treated cells via p53 knock-down suggest that p53 is likely upstream of the NFκB-induced survival response.

## Supplementary Information

**Additional file 1: Table S1.** Cisplatin and INI-43 concentrations used in the combination index determination experiment. Cells were treated with cisplatin only, INI-43 only or a combination of the two using the concentrations indicated below.

**Additional file 2: Supplementary Figure 1.** INI-43 washout experiment showing that short exposure to INI-43 is sufficient to enhance cisplatin-induced cell death. SiHa (A) and HeLa (B) cells were exposed to INI-43 for 2 h, after which cisplatin was added with INI-43 still present (no washout), immediately following INI-43 removal (washout 1), or 2 h after INI-43 removal (washout 2). Cell viability was measured after 48 h using the MTT assay (*p < 0.05). **Supplementary Figure 2.** Full length blots for Fig. 1c. **Supplementary Figure 3.** A. Full length blots for Fig. 3a. B. Full length blots for Fig. 3d. **Supplementary Figure 4.** A. Full length blots for Fig. 4a., B. Full length blots for Fig. 4c. C. Full length blots for Fig. 4e. **Supplementary Figure 5.** A. Full length blots for Fig. 5a, B. Full length blots for Fig. 5b, C. Full length blots for Fig. 5f, D. Full length blots for Fig. 5g. **Supplementary Figure 6**. A. Full length blots for Fig. 6e. B. Full length blots for Fig. 6g.

## Data Availability

Data sharing is not applicable to this article as no datasets were generated or analysed during the current study.

## Abbreviations

Kpnβ1: Karyopherin beta 1
Crm1: Chromosome Region Maintenance 1
CT: Combination treatment
IC_50_: Half inhibitory concentration
CI: Combination index
Fa: Fraction affected
T_1/2_: Half-life
CHX: Cycloheximide
si-Kpnβ1: Kpnβ1-targeting siRNA
si-ctrl: Control siRNA
Fc (Nu/Cy): Nuclear fluorescence relative to cytoplasmic fluorescence
XIAP: X-Chromosome Linked Inhibitor of Apoptosis
γH2AX: Phosphorylated Histone 2AX
