# Supplementary material for: Inhibition of Kpnβ1 mediated nuclear import enhances cisplatin chemosensitivity in cervical cancer
Source: BMC Cancer. 2021 Feb 2;21:106. doi: 10.1186/s12885-021-07819-3 (PMC7852134; doi:10.1186/s12885-021-07819-3)
Supplement: Supplementary file 1 — Additional file 1: Table S1. Cisplatin and INI-43 concentrations used in the combination index determination experiment. Cells were treated with cisplatin only, INI-43 only or a combination of the two using the concentrations indicated below. [file 12885_2021_7819_MOESM1_ESM.docx]

**TABLES**

Table S1. Cisplatin and INI-43 concentrations used in the combination index determination experiment. Cells were treated with cisplatin only, INI-43 only or a combination of the two using the concentrations indicated below.

| **Cisplatin (μM)** | **INI-43 (μM)** | | |
| --- | --- | --- | --- |
|  | **1INI-43:3 cisplatin** | **1INI-43:4 cisplatin** | **1INI-43:5 cisplatin** |
| **3** | 1.00 | 0.75 | 0.60 |
| **7.5** | 2.50 | 1.88 | 1.50 |
| **15** | 5.00 | 3.75 | 3.00 |
| **22.5** | 7.50 | 5.63 | 4.50 |
| **30** | 10.00 | 7.50 | 6.00 |
| **45** | 15.00 | 11.25 | 9.00 |
