# Supplementary material for: Inhibition of Kpnβ1 mediated nuclear import enhances cisplatin chemosensitivity in cervical cancer
Source: BMC Cancer. 2021 Feb 2;21:106. doi: 10.1186/s12885-021-07819-3 (PMC7852134; doi:10.1186/s12885-021-07819-3)
Supplement: Supplementary file 2 — Additional file 2: Supplementary Figure 1. INI-43 washout experiment showing that short exposure to INI-43 is sufficient to enhance cisplatin-induced cell death. SiHa (A) and HeLa (B) cells were exposed to INI-43 for 2 h, after which cisplatin was added with INI-43 still present (no washout), immediately following INI-43 removal (washout 1), or 2 h after INI-43 removal (washout 2). Cell viability was measured after 48 h using the MTT assay (*p < 0.05). Supplementary Figure 2. Full length blots for Fig. 1c. Supplementary Figure 3. A. Full length blots for Fig. 3a. B. Full length blots for Fig. 3d. Supplementary Figure 4. A. Full length blots for Fig. 4a., B. Full length blots for Fig. 4c. C. Full length blots for Fig. 4e. Supplementary Figure 5. A. Full length blots for Fig. 5a, B. Full length blots for Fig. 5b, C. Full length blots for Fig. 5f, D. Full length blots for Fig. 5g. Supplementary Figure 6. A. Full length blots for Fig. 6e. B. Full length blots for Fig. 6g. [file 12885_2021_7819_MOESM2_ESM.docx]

**Supplementary figure 1. INI-43 washout experiment showing that short exposure to INI-43 is sufficient to enhance cisplatin-induced cell death.** SiHa (A) and HeLa (B) cells were exposed to INI-43 for 2 hours, after which cisplatin was added with INI-43 still present (no washout), immediately following INI-43 removal (washout 1), or 2 hours after INI-43 removal (washout 2). Cell viability was measured after 48 hours using the MTT assay (*p<0.05).

**Supplementary figure 2. Full length blots for Fig. 1C.**

**Supplementary figure 3. A. Full length blots for Fig. 3A. B. Full length blots for Fig. 3D.**

**Supplementary figure 4. A. Full length blots for Fig. 4A., B. Full length blots for Fig. 4C. C. Full length blots for Fig. 4.E.**

**Supplementary figure 5. A. Full length blots for Fig. 5A., B. Full length blots for Fig. 5B., C. Full length blots for Fig. 5F., D. Full length blots for Fig. 5.G.**

**Supplementary figure 6. A. Full length blots for Fig. 6E. B. Full length blots for Fig. 6G.**
